# Rice OsBRCA2 Is Required for DNA Double-Strand Break Repair in Meiotic Cells

**DOI:** 10.3389/fpls.2020.600820

**Published:** 2020-11-16

**Authors:** Ruifeng Fu, Chong Wang, Hongyu Shen, Jie Zhang, James D. Higgins, Wanqi Liang

**Affiliations:** ^1^Joint International Research Laboratory of Metabolic & Developmental Sciences, Shanghai Jiao Tong University–University of Adelaide Joint Centre for Agriculture and Health, State Key Laboratory of Hybrid Rice, School of Life Sciences and Biotechnology, Shanghai Jiao Tong University, Shanghai, China; ^2^Department of Genetics and Genome Biology, University of Leicester,Leicester, United Kingdom

**Keywords:** meiosis, homologous recombination, BRCA2, DMC1, RAD51

## Abstract

The mammalian *BREAST CANCER 2* (*BRCA2*) gene is a tumor suppressor that plays a crucial role in DNA repair and homologous recombination (HR). Here, we report the identification and characterization of *OsBRCA2*, the rice orthologue of human *BRCA2*. *Osbrca2* mutant plants exhibit normal vegetative growth but experience complete male and female sterility as a consequence of severe meiotic defects. Pairing, synapsis and recombination are impaired in *osbrca2* male meiocytes, leading to chromosome entanglements and fragmentation. In the absence of OsBRCA2, localization to the meiotic chromosome axes of the strand-invasion proteins OsRAD51 and OsDMC1 is severely reduced and *in vitro* OsBRCA2 directly interacts with OsRAD51 and OsDMC1. These results indicate that OsBRCA2 is essential for facilitating the loading of OsRAD51 and OsDMC1 onto resected ends of programmed double-strand breaks (DSB) during meiosis to promote single-end invasions of homologous chromosomes and accurate recombination. In addition, treatment of *osbrca2-1* seedlings with mitomycin C (MMC) led to hypersensitivity. As MMC is a genotoxic agent that creates DNA lesions in the somatic cells that can only be repaired by HR, these results suggest that OsBRCA2 has a conserved role in DSB repair and HR in rice.

## Introduction

Cellular DNA of living organisms experience DNA damage caused by exogenous and endogenous factors. DNA double-strand breaks (DSBs) are one of the most cytotoxic DNA lesions, as even one single unrepaired or misrepaired DSB will lead to genomic rearrangements and genome instability (Bennett et al., 1993; Sandell and Zakian, 1993). Non-homologous end joining (NHEJ) and homologous recombination (HR) are the two main pathways involved in the repair of DSBs (Bétermier et al., 2014; Guirouilh-Barbat et al., 2014). In the NHEJ pathway, DNA broken ends are repaired by rapidly ligating the two DNA ends with little to no DNA end processing. In contrast to the error-prone NHEJ pathway, the HR pathway uses an intact homologous DNA sequence as the repair template to ensure faithful repair. In addition to accidental mitotic DSBs, HR is indispensable for repairing programmed meiotic DSBs that are intentionally produced by a DNA topoisomerase VI-like complex at the beginning of meiotic prophase I (Fu et al., 2016; Fayos et al., 2020).

DSB repair *via* HR is initiated by 5' to 3' resection of the DNA ends with the creation of 3' single-stranded (ssDNA) overhangs, which are then coated by replication protein A (RPA) to prevent from degradation and forming secondary structures (Iftode et al., 1999). Subsequently, RPA proteins are replaced by recombinases, which are essential for strand invasion and DNA homology searching. In bacteria, RecA plays a pivotal role in strand exchange between homologous DNA molecules (Roca and Cox, 1997). In eukaryotic cells, two of RecA homologs, RAD51 and meiotic specific DMC1, possess the activity to catalyze the pairing of homologous DNA sequences and strand exchange reaction to fulfill the homology directed DSB repair (San Filippo et al., 2008). RAD51 uses the intact sister chromatid as the template to repair DSBs; while in meiotic cells, DMC1 employs homologous chromosomes as templates with the aid of RAD51 (Cloud et al., 2012; Kurzbauer et al., 2012; Da Ines et al., 2013), that may result in the formation of crossover (CO) between homologous chromosomes.

The formation of RAD51/DMC1 nucleoprotein filaments is considered as a rate-limiting process of HR and is mediated by the RAD51/DMC1 loader BRCA2. The *BRCA2* gene was first identified in humans (Wooster et al., 1995), whose mutations have been reported to be the cause of hereditary breast cancers and confer a high risk to many other cancers (Hall et al., 1990). *HsBRCA2* encodes a protein with 3418 amino acids, consisting of several functional domains, including eight BRC repeats, a phenylalanine-proline-proline (PhePP) motif, a DNA-binding domain (DBD) containing a helical rich region, three oligonucleotide/oligosaccharide binding (OB) folds, and a C-terminal TR2 domain (Dray et al., 2006; Thorslund et al., 2007; Seeliger et al., 2012). Biochemical studies revealed that BRCA2 is loaded onto both ends of the double-stranded DNA (dsDNA) and single-stranded DNA (ssDNA), leading to the formation of ssDNA-dsDNA junctions (Yang et al., 2002, 2005). BRCA2 interacts with RAD51 and DMC1 *via* its BRC repeats and promotes their loading onto the ends of ssDNA covered by RPA (Wong et al., 1997; Chen et al., 1998). Besides, the C-terminal TR2 domain of BRCA2 interacts with multimeric RAD51 to stabilize the RAD51 nucleofilaments (Davies and Pellegrini, 2007; Esashi et al., 2007). Furthermore, BRCA2 interacts with multiple partner proteins to assist in RAD51/DMC1 loading and assembly, such as DSS1 (Marston et al., 1999), USP11 (Schoenfeld et al., 2004), BCCIP (Lu et al., 2005) FANCD2 (Hussain et al., 2003, 2004; Wang et al., 2004), FANCG (Hussain et al., 2003, 2004; Wang et al., 2004) and PALB2/FANCN (Xia et al., 2006; Sy et al., 2009; Zhang et al., 2009a,b; Orthwein et al., 2015). Thus, BRCA2 functions as a master regulator in promoting HR-mediated DSB repair to maintain genome integrity.

Homologs of tumor suppressor HsBRCA2 have been identified in various eukaryotes including plants but are absent from archaea, bacterial, and yeast. Although highly variable in protein size and the number of BRC repeats, the ability of BRCA2 homologs to associate with recombinases is conserved in several organisms. *Arabidopsis* is the only known organism that has two BRCA homologs (Pfeffer et al., 2017). In mammals, *BRCA2* is essential for survival; its deficiency results in embryonic lethality. However, double knockouts of *AtBRCA2A/B* does not affect normal vegetative growth but causes severe abnormalities in meiosis, including defective homologous chromosome pairing and synapsis, chromosome entanglement, and fragmentation, leading to both male and female sterility (Seeliger et al., 2012). The fact that the *brca2* phenotype is alleviated by the *spo11* mutation indicates that its meiotic chromosomal instability is caused by failure in repairing programmed DSBs (Seeliger et al., 2012). AtBRCA2A/B has been proven to be able to interact with AtRAD51 and AtDMC1 *in vitro* and *in vivo* (Abe et al., 2009; Wang et al., 2010b). In *atbrca2* double mutants, AtRAD51 and AtDMC1 foci could not be detected in meiotic cells (Seeliger et al., 2012). Additionally, the *atbrac2a* single mutant and *atbrca2* double mutants display hypersensitivity to the genotoxic agent mitomycin C (MMC) and defects in somatic HR (Abe et al., 2009; Wang et al., 2010b). These data indicate that AtBRCA2 plays a conserved role in HR by recruiting RAD51 and DMC1, essential for HR mediated DSB repair in both meiotic and somatic cells.

Compared with the well-documented BRCA2 functions in animals and fungi, much less is known about the role of BRCA2 homologs in plants. Currently, only *Arabidopsis BRCA2* has been functionally characterized and the role of the *BRCA2* orthologs in other plants remains unknown. In this study, we identified and characterized the *BRCA2* ortholog in *Oryza sativa*. Our results reveal that *OsBRCA2* is essential for promoting HR and chromosome synapsis, as well as maintaining the genome stability in meiotic cells. Furthermore, *osbrca2* is hypersensitive to the DNA damage agent MMC. Notably, we demonstrated that OsBRCA2 is able to facilitate OsRAD51 and OsDMC1 loading onto the chromosomes. This study provides further evidence to support the crucial function of the BRCA2 in HR-mediated DSBs repair pathway in plants.

## Materials and Methods

### Plant Materials, Growth Conditions, and Molecular Cloning of *OsBRCA2*

Rice (*Oryza sativa*) plants in the 9522 background (*O. sativa ssp japonica*) were grown in the paddy field of Shanghai Jiao Tong University under natural growth condition. The mapping population was collected from the F2 progenies that were generated from a cross between *osbrca2-1* mutant and Guang-Lu-Ai4 (*O. sativa* ssp *indica*). Primary-mapping of *OsBRCA2* was performed using bulked segregated analysis (Liu et al., 2005). Three BRCA2 alleles, including *osbrca2-2*, *osbrca2-3*, and *osbrca2-4*, were created using the CRISPR-Cas9 system kindly provided by Professor Jiankang Zhu. The sgRNA-Cas9 plant expression vectors were constructed as previously described (Wang et al., 2017). Primers used for fine mapping, constructing sgRNA-Cas9 plant expression vectors and verifying transgenic plants are listed in Supplementary Table S1.

### Characterization of Mutant Plant Phenotypes

Rice plants, spikelets, and anthers at heading stage were photographed with digital camera (Nikon, E995) or under a stereoscope (Leica, M205A). The pollen viability assay was performed by 1% (w/v) I_2_-KI staining. The transverse sections of anthers were obtained according to the method previously published (Li et al., 2006).

### Antibody Production

The OsBRCA2 and OsRAD51 polyclonal antibodies used in this study were prepared by Abclonal (Wuhan, China). A 600-bp DNA fragment encoding a 200-amino acid peptide of OsBRCA2 (residues 251-451) and a coding sequence of OsRAD51 encoding a 200-amino acid peptide of OsRAD51 (residues 1-200) were amplified from the rice anther cDNA and cloned into the protein expression vector pET-32a (GE) respectively. The recombinant proteins expressed in *Escherichia coli* BL_21_DE_3_ (Novagen) were purified and used to produce rabbit polyclonal antibodies. The polyclonal antibodies against OsREC8, γH2AX, COM1, RPA1c, RPA2c, RAD51C, DMC1, PAIR2, PAIR3, and ZEP1 have been described in previous studies (Fu et al., 2016; He et al., 2016).

### FISH Analysis and Immunolocalization Assays

Fresh panicles containing male meiocytes were harvested and fixed with Carnoy’s solution (ethanol: glacial acetic 3:1, v/v). The preparation and DAPI staining of meiotic chromosomes were performed as previously described (He et al., 2016). Fluorescent *in situ* hybridization (FISH) assay was performed as described (Cheng, 2013). The sequences of the centromere and 5*S* rDNA FISH probes were designed and labeled as described (Mizuno et al., 2006; Zhang et al., 2019). Images were captured with an Eclipse Ni-E microscope and NIS elements software (Nikon). Immunolocalization assays were performed as described in previous studies (Cheng, 2013; He et al., 2016; Wang et al., 2017). Fresh panicles containing meiocytes were fixed in 4% (w/v) paraformaldehyde for 30 min at room temperature and then washed three times with 1 × PBS (pH 7.4). Anthers were squashed on a slide with 1 × PBS solution (pH 7.4) and soaked in liquid nitrogen. After removing the cover slips quickly with a blade, the slides were dehydrated through an ethanol series (70, 90, and 100%). Different antibody combinations diluted to 1:200 in TNB buffer (0.1 M Tris-HCl, pH 7.5, 0.15 M NaCl, and 0.5% blocking reagent) were added to the slides and then incubated in a humid chamber at 37°C for 2 h. After three rounds of washing in 1 × PBS, goat anti-rabbit antibody (Alexa Fluor® 555, Life Technologies, 1:500) and goat anti-mouse antibody (Dylight 488, Abbkine, 1:500) were added to the slides, and then incubated in a humid chamber at 37°C for 1 h. Finally, the slides were counterstained with DAPI after three rounds of washing in 1 × PBS. All fluorescence images were photographed and processed using an Eclipse Ni-E microscope (Nikon) with NIS-Elements Advanced Research software at the same parameter level to avoid the interference of multi-factor. Each channel keeps the same exposure values to capture red-green channel images as presented in visual results. For dot-like foci signals, image deconvolution will be further performed using “Mexican Hat” process to improve signal-to-noise ratio. The number of dot-like fluorescent foci signals was counted using ImageJ 1.52 software (Collins, 2007).

### Yeast Two-Hybrid Assay

The full-length cDNAs of *OsBRCA2* and a 2760-bp cDNA fragment encoding the six BRC domains of OsBRCA2 were amplified from rice anther cDNA and cloned into the pGBKT7 vector (Clontech) separately. The full-length cDNAs of *OsDMC1A*, *OsDMC1B*, *OsRAD51A1*, *OsRAD51A2*, was amplified from rice anther cDNA and cloned into the pGADT7 vector (Clontech), respectively. Subsequently, yeast two-hybrid (Y2H) assays were performed according to the manufacturer’s instructions (Clontech). Primers used for cDNA amplification and vector construction are listed in Supplementary Table S1.

### qRT-PCR Assay

Total RNA from wild-type tissues was isolated using the Trizol Reagent Kit (Invitrogen) according to the manufacturer’s protocol. The rice anther development stage was defined as previously described (Zhang et al., 2011). Roots, shoots, and leaves were collected from 30-day-old seedlings and glumes were collected from stage 8 spikelets. An equal amount total RNA per sample was used to synthesize cDNA using Primescript™ RT reagent kit with genomic DNA eraser (Takara). The qRT-PCR analysis was performed according to the previous report (Fu et al., 2014). The rice *Actin* gene was used as the internal control, and primers used for qRT-PCR are listed in Supplementary Table S1. All reactions were performed in three independent biological replicates with three technical repeats each for statistical analysis. The gene expression was calculated by the 2^-ΔΔCt^ method (Livak and Schmittgen, 2001).

### MMS and MMC Sensitivity Test

The genotoxic agents sensitivity tests were performed using the methods as described by Chang et al. (2009) and modified as follows. Husked and surface-sterilized seeds of the heterozygous *OsBRCA2-1*^+/−^ and wild type were germinated and grown on 1/2 MS (Murashige and Skoog) medium for 5 days. Subsequently, these seedlings were divided into three portions used for three independent biological repeats. Then seedlings were transferred to 1/2 MS (Murashige and Skoog) medium supplemented with 0.3% phytagel and with concentrations 0 μl/L to 150 μl/L of methylmethane sulphonate (MMS; Sigma-Aldrich, St. Louis, MO, United States) or 0–300 μg/ml MMC (Sangon, Shanghai, China). A total of 90 seedlings in each treatment arranged in six replications of 15 seedlings per plastic pot were placed in a light incubator. After 13 days treatment, the genotypes were verified by PCR and the height of seedlings was measured. Student’s *t* tests were performed for comparing the data differences, *p* < 0.05 was considered to be significant. ^*^ represented *p* < 0.05, and ^**^ represented *p* < 0.01.

### Accession Numbers

Sequence data from this article can be found in the GenBank/EMBL data libraries under the following accession numbers: *OsBRCA2* (*Os01g0164800*, *Os01g0164900*), *OsRAD51A1* (*Os11g0615800*), *OsRAD51A2* (*Os12g0497300*), *OsDMC1A* (*Os12g0143800*), *OsDMC1B* (*Os11g0146800*), *OsActin* (*Os03g0718100*), *AtBRCA2A* (NP_191913.3), *AtBRCA2B* (NP_195783.3), and *HsBRCA2* (*CAA64484.1*).

## Results

### Identification of the *osbrca2-1* Mutant

**Figure 1:**
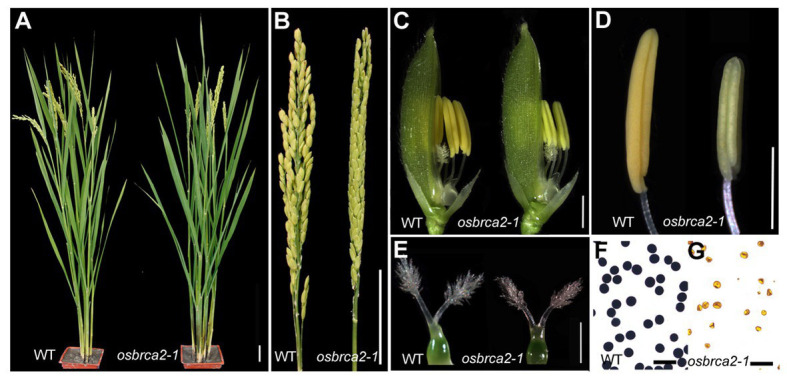
Phenotypic comparison between wild-type and *osbrca2-1* mutant. **(A)** Wild-type and *osbrca2-1* plants after heading. **(B)** Wild-type and *osbrca2-1* panicles showing fertile and infertile grains, respectively. **(C)** Wild-type and *osbrca2-1* spikelets after removing lemma. **(D)** Wild-type and *osbrca2-1* pistils at stage 12. **(E)** Wild-type (left) and *osbrca2-1* (right) anthers. **(F)** I_2_-KI staining of wild-type and **(G)** *osbrca2-1* pollen grains at mature stage. Bars = 5 cm in **(A)** and **(B)**, 5 mm in **(C)**, 1 mm in **(D)** and **(E)**, and 100 μm in **(F)** and **(G)**.

To isolate genes essential for rice fertility, we screened for sterile mutants from a rice (*O. sativa* ssp *japonica cultivar*, 9522) mutant library (Chen et al., 2006) and identified a completely sterile mutant *osbrca2-1*. During vegetative developmental stage, the *osbrca2-1* plant grew normally as wild-type plants (Figure 1A). However, during the reproductive stage, *osbrca2-1* had small, pale-yellow stamens (Figures 1C,E) that could not produce mature pollen grains or seeds (Figures 1B,F,G). Female fertility of *osbrca2-1* was also abnormal, shown by the smaller pistil (Figure 1D) and inability to set seeds when pollinated with wild-type pollen. Progeny from heterozygote plants segregated 298 fertile plants and 92 sterile plants (3:1, *χ*^2^ = 0.4137 < *χ*^2^_0.05, 1_ = 3.84), indicating that *osbrca2-1* was a single recessive mutation.

**Figure 2:**
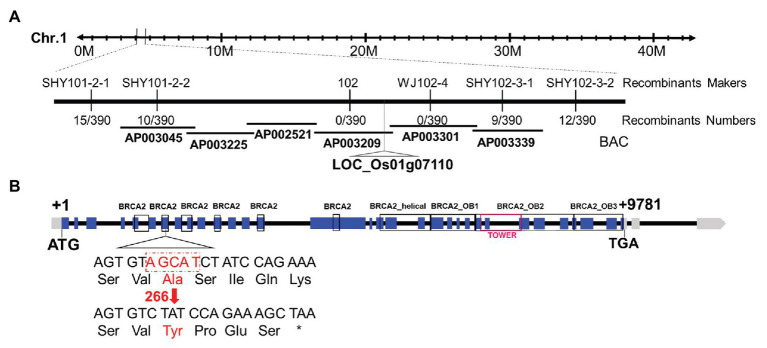
Molecular characterization of *osbrca2-1*. **(A)** Fine mapping of *osbrca2-1* on chromosome 1. Names and positions of the markers are indicated. **(B)** A schematic representation of 32 exons and 31 introns of LOC_Os01g07110. The +1 indicates the putative starting nucleotide of translation, and the stop codon (TGA) is +9781. Blue boxes indicate exons, and intervening lines indicate introns. The deletion site in *osbrca2-1* is shown (red arrow).

A map-based cloning strategy was exploited to identify the mutated gene by using 298 mutants from an F2 mapping population. The mutated locus was located between two Indel molecular markers named SHY101-2-2 and SHY102-3-1 on chromosome 1 (Figure 2A). Whole-genome sequencing revealed a 5 bp deletion in the seventh exon of the candidate gene (LOC_Os01g07110), leading to a frame shift and a premature stop codon (Figure 2B). Sequence analysis showed that LOC_Os01g07110 encodes a protein sharing high sequence similarity with the human breast cancer susceptibility gene 2 (*HsBRCA2*; Thorslund and West, 2007), thus we named this gene as *OsBRCA2*. The predicted OsBRCA2 protein was 1575 amino acids in length and contained six putative BRC repeats, one OsBRCA2_helical, one OsBRCA2_OB1 domain, one OsBRCA2_OB2 domain containing one TOWER domain and one OsBRCA2_OB3 domain (Figure 2B).

To confirm that the mutation in *OsBRCA2* was responsible for the sterile phenotype of *osbrca2-1* mutant, three independent alleles *osbrca2-2*, *osbrca2-3*, *osbrca2-4* were generated using the CRISPR/Cas9 system. *Osbrca2-2* and *osbrca2-4* had a T insertion in the first BRC repeat and BRCA2_OB1 domain, which caused frame shift from 186th aa and 1153th aa respectively. *Osbrca2-3* had a 2 bp (GT) deletion in the third BRC repeat that caused a frame shift from 332th aa and premature translation termination (Supplementary Figure S1A). All of the *osbrca2-2, osbrca2-3*, and *osbrca2-4* homozygous plants were sterile (Supplementary Figures S1C,G,K) and displayed similar meiotic defects (see below), confirming that the sterility that occurred in *osbrca2-1* was the consequence of mutation in *OsBRCA2*.

### Expression Pattern of *OsBRCA2*

The spatial and temporal expression pattern of *OsBRCA2* was investigated by qRT-PCR analysis. *OsBRCA2* was highly expressed in the anther before entry into meiosis and then declined after meiosis. *OsBRCA2* was also highly expressed in leaves and weakly expressed in shoot, glume, and root (Supplementary Figure S2). The results showed that the transcripts of *OsBRCA2* were extensively expressed not only in reproductive organs but also in vegetative organs.

**Figure 3:**
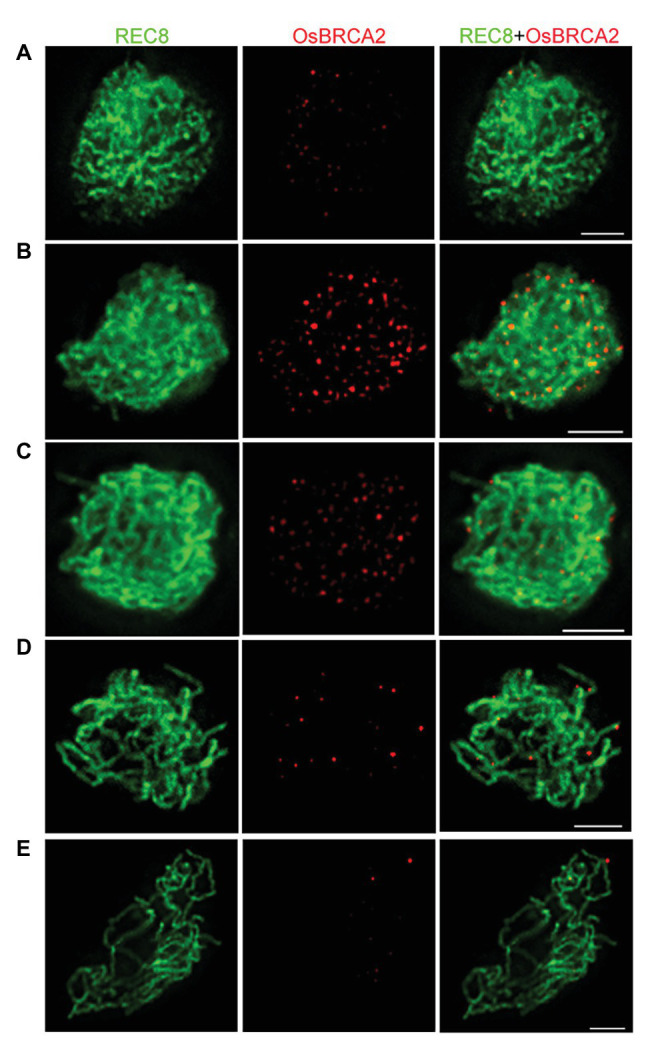
Dual immunolocalization assay of OsBRCA2 (red) and REC8 (green) in the wild-type meiocyte cells. **(A)** Late leptotene. **(B)** Zygotene. **(C)** Late zygotene. **(D)** Early pachytene. **(E)** Late pachytene. Bars = 5 μm.

To further define the spatiotemporal localization of OsBRCA2 during meiosis, dual immunolocalization assays utilizing antibodies raised against OsREC8 and OsBRCA2 were performed. In wild type, OsBRCA2 foci were first observed at late leptotene (Figure 3A). The number of OsBRCA2 foci dramatically increased and peaked at zygotene (Figure 3B). After that, OsBRCA2 foci decreased and very few signals could be detected at late pachytene (Figures 3C–E). No OsBRCA2 signal can be detected in *osbrca2-1* lines, indicating that the OsBRCA2 antibody is specific (Supplementary Figure S3).

### Meiosis Is Impaired in *osbrca2* Mutants

To identify the developmental defects responsible for *osbrca2-1* male fertility, transverse sections of *osbrca2-1* and wild-type anthers were performed. There were no distinguishable differences between wild type and *osbrca2-1* before meiosis (Zhang et al., 2011). The pollen mother cells (PMCs) of wild type could produce dyads and tetrads, and released microspores normally. While PMCs of *osbrca2-1* produced polyads at the end of meiosis, and generated severely distorted and shrunken microspores (Supplementary Figure S4).

**Figure 4:**
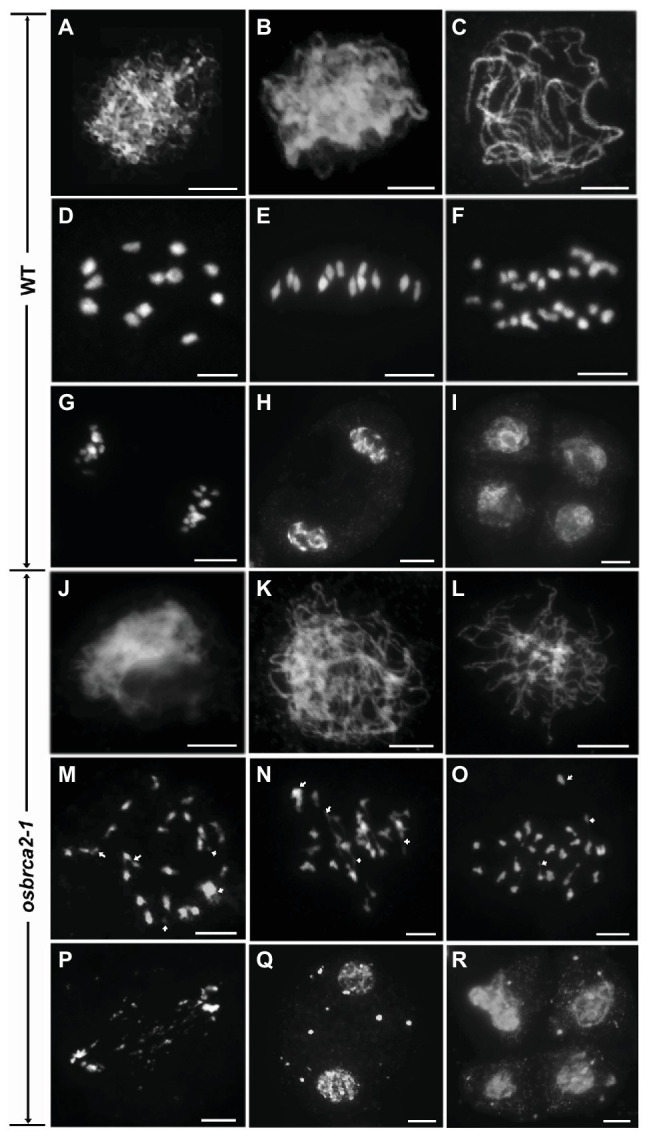
Meiotic chromosome behavior of male meiocytes in the wild-type and *osbrca2-1*. Chromosome behavior of male meiocytes of wild type **(A–I)** and *osbrca2-1* **(J–R)** at various stages. **(A,J)** Leptotene. **(B,K)** Zygotene. **(C,L)** Pachytene. **(D,M)** Diakinesis. **(E,N)** Metaphase I. **(F,O)** Anaphase I. **(G,P)** Telophase I. **(H,Q)** Dyad. **(I,R)** Tetrad. Arrows indicated the abnormal chromosomes. Bars = 5 μm.

Anther transverse sections indicated that the complete sterility of *osbrca2-1* may be caused by meiotic defects. To investigate the roles of OsBRCA2 in meiosis, we performed 4', 6-diamidino-2-phenylindole (DAPI) staining to analyze the chromosome behavior in wild-type and *osbrca2-1* male meiocytes. In wild type, the chromosomes completed the DNA replication process and began to condense into visible strands at leptotene (Figure 4A). During zygotene, the chromosomes continued to condense and pair as well as initiating synapsis (Figure 4B). At pachytene, synapsis and recombination occurred normally within homologous chromosomes (Figure 4C). At diplotene, the synaptonemal complexes (SCs) began to disassemble with the formation of chiasma that physically linked homologous chromosomes together. Twelve bivalents formed at diakinesis (Figure 4D) then aligned onto the equatorial plate at metaphase I (Figure 4E). At anaphase I, homologous chromosomes began to separate (Figure 4F) and migrated in opposite directions at telophase I (Figure 4G). Dyads and tetrads were formed at the end of meiosis I and II, respectively (Figures 4H,I). In *osbrca2-1*, chromosome behavior appeared to show no obvious differences from leptotene to zygotene when compared with wild type (Figures 4J,K). However, at pachytene, the chromosomes appeared only partially synapsed (Figure 4L). At diakinesis, irregularly shaped univalents, a small number of chromosome fragments and chromosome bridges were observed (Figure 4M). At metaphase I, chromosomes were entangled and could not align along the equatorial plate; chromosome bridges became more conspicuous (Figure 4N). At anaphase I, chromosomes asynchronously separated to the two opposite poles, as well as non-segregating chromosome fragments inside the nucleus (Figure 4O). At telophase I, lagging chromosome fragments could still be seen distributed between two newly formed nuclei (Figure 4P), leading to the formation of micronuclei in cells at the dyad and tetrad stage (Figures 4Q,R). We also monitored *osbrca2-2*, *osbrca2-3*, and *osbrca2-4* meiotic progression by DAPI staining (Supplementary Figures S1D–F,H–J,L–N). The meiotic defects in these alleles were similar to those observed in *osbrca2-1*. These results indicate that the sterility of *osbrca2* mutants was caused by the failure of DNA repair by HR and genome fragmentation during meiosis.

### OsBRCA2 Is Essential for Homologous Chromosome Pairing and Synaptonemal Complex Formation

**Figure 5:**
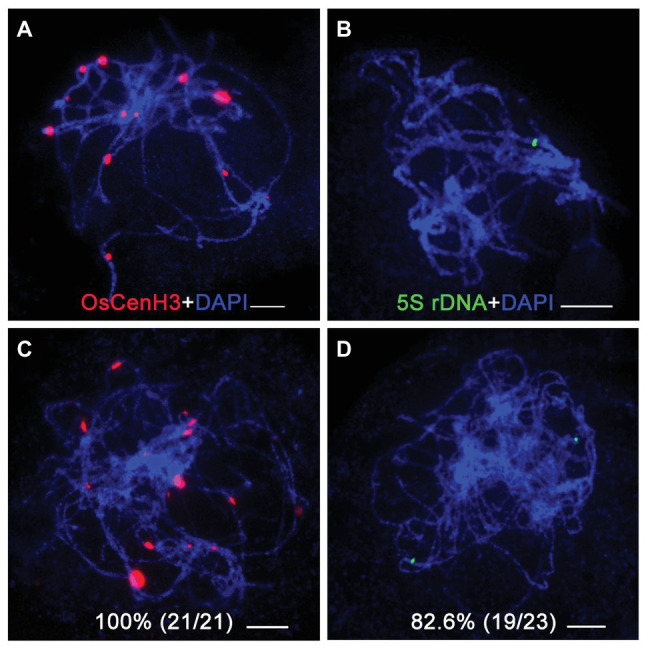
Homologous chromosomes paring is impaired in *osbrca2-1* mutant. Homologous pairing analysis by fluorescent *in situ* hybridization (FISH) using OsCenH3 (red) and 5*S* rDNA (green) probes in wild-type **(A,B)** and *osbrca2-1* mutant **(C,D)**. Chromosomes (blue) are stained with DAPI. Percentage represents the number of abnormal cells, *n* = 21 and 23, respectively. Bars = 5 μm.

To investigate homologous chromosome pairing in the *osbrca2-1* mutant, we performed fluorescent *in situ* hybridization (FISH) analysis using a centromere specific probe OsCenH3 and the 5*S* rDNA probe specifically distributed on the short arm of chromosome 11 (Zhang et al., 2005). In wild type, there were 12 OsCenH3 signals observed at pachytene (Figure 5A), but an average of 17 signals (*n* = 21, range 15–20) were detected in the *osbrca2-1* mutant (Figure 5C). In the wild type, only one 5*S* rDNA signal can be detected (Figure 5B), which indicates full homologous chromosome pairing and synapsis. However, in the *osbrca2-1* mutant, two totally separated 5*S* rDNA signals were observed in most nuclei (82.6%, *n* = 23; Figure 5D), suggesting that homologous chromosome pairing is defective.

**Figure 6:**
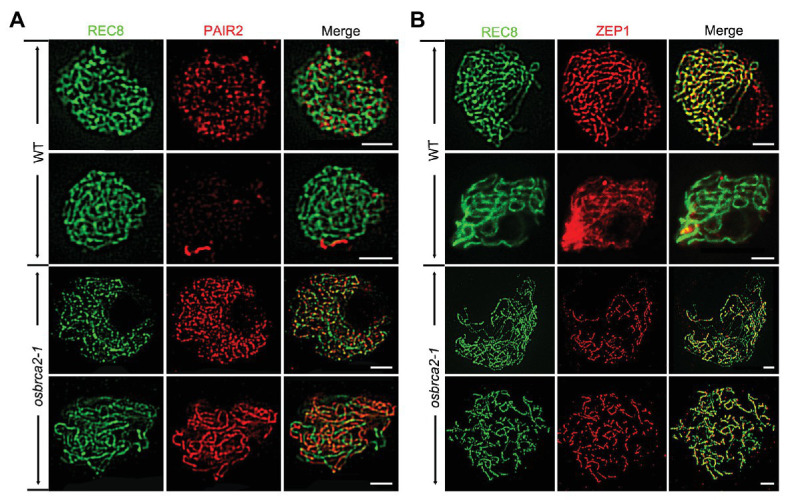
Synapsis complex is not correctly installed in *osbrca2-1* mutant. Dual immunolocalization of PAIR2 (red) **(A)**, ZEP1 (red) **(B)** and OsREC8 (green) in wild-type and *osbrca2-1* PMCs. Bars = 5 μm.

To further understand whether synaptonemal complex formation was defective in *osbrca2-1*, we conducted immunolocalization analysis using SC related proteins: OsREC8, PAIR2, PAIR3 and ZEP1 (Nonomura et al., 2006; Wang et al., 2010a, 2011; Shao et al., 2011). OsREC8 is a meiotic cohesin complex component and required for sister chromatid cohesion, axial element (AE) formation and homologous pairing (Shao et al., 2011). In *osbrca2-1* male meiocytes, OsREC8 localized normally as that in wild-type male meiocytes and was used as a marker for further immunolocalization analysis (Figure 4). PAIR2 associated with axial elements (AEs) at leptotene and zygotene and then disassociated from the AEs of arm regions when homologous chromosomes fully synapsed (Nonomura et al., 2006). From zygotene to pachytene, PAIR2 signals became weaker and discontinuous on wild-type meiotic chromosomes (*n* = 10, Figure 6A). However, in *osbrca2-1*, PAIR2 foci did not disappear at pachytene (*n* = 13, Figure 6A). As an axis-associated protein, PAIR3 is also essential for SC assembly, which might provide a platform for other recombination elements, such as PAIR2 (Wang et al., 2011). By contrast, there was no obvious difference in PAIR3 localization between *osbrca2-1* and wild type (*n* = 10, Supplementary Figure S5). *ZEP1* encodes the transverse filament protein of the rice SC (Wang et al., 2010a). In wild type, ZEP1 formed punctate foci during zygotene and then elongated into continuous linear signals and aligned perfectly along the entire chromosome when homologous fully synapsed at pachytene (*n* = 11, Figure 6B). While in the *osbrca2-1* mutant, only punctate or short discontinuous linear ZEP1 signal was observed even at late pachytene (*n* = 12, Figure 6B). These observations suggest that SC extension was severely interrupted in *osbrca2-1* meiocytes.

### OsBRCA2 Is Essential for Crossover Formation by Mediating Recruitment of OsRAD51 and OsDMC1

In many species, homologous chromosome recombination requires the formation and repair of double strand breaks (DSBs; Inagaki et al., 2010). Unrepaired or incorrectly repaired DSBs will lead to the formation of chromosomal aberrations such as broken chromosomes and/or gross chromosomal rearrangements (Pastink et al., 2001). In order to determine whether the chromosome fragments were caused by unrepaired or incorrectly repaired DSBs, we generated *osbrca2-1 ossds* double mutant. SDS is a meiosis specific cyclin-like protein, which is essential for meiotic DSB formation in rice (Wu et al., 2015). In an *ossds* single mutant, due to defective homologous chromosome pairing and synapsis, only univalents were detected at diakinesis and metaphase I (Supplementary Figures S6C1,C2), and no chromosome fragmentation was observed (Supplementary Figures S6C1–C6). In the *osbrca2-1 ossds* double mutants, no chromosome entanglements or fragments were observed, similar to that in the *ossds* single mutant (Supplementary Figures S6D1–D6). The chromosome abnormalities in *osbrca2-1* were totally suppressed by loss of the *OsSDS* function. Therefore, we conclude that unrepaired DSBs are responsible for the chromosome entanglement and fragmentation generated in *osbrca2-1*.

To further determine whether OsBRCA2 is involved in DSB formation or processing, we applied antibodies raised against γH2AX, COM1, RPA1c, RPA2c, OsRAD51A, and OsDMC1, to perform an immunolocalization analysis on wild-type and *osbrca2-1* male meiocytes. γH2AX is the phosphorylated form of Histone 2A.X and is a reliable marker for detection of DSB sites (Hunter et al., 2001; Mahadevaiah et al., 2001). γH2AX immunolocalization showed no significant difference between wild type (200.8, *n* = 35) and *osbrca2-1* (212.2, *n* = 26; Supplementary Figure S7A), indicating that OsBRCA2 is dispensable for DSB initiation. It had been reported that COM1, RPA1c and RPA2c were required for DSB end-processing and 3' single strand invasion after DSB generation (Ji et al., 2012; Li et al., 2013; Tang et al., 2014). The localization of these proteins was also indistinguishable in *osbrca2-1* (154.3, *n* = 30; 161.0, *n* = 22; and 211.8, *n* = 26, respectively) when compared with that in wild type (163.7, *n* = 34; 152.9, *n* = 22; and 211.2, *n* = 27, respectively; Supplementary Figures S7B–D). These results indicated that OsBRCA2 is not required for DSB production or resection end-processing.

**Figure 7:**
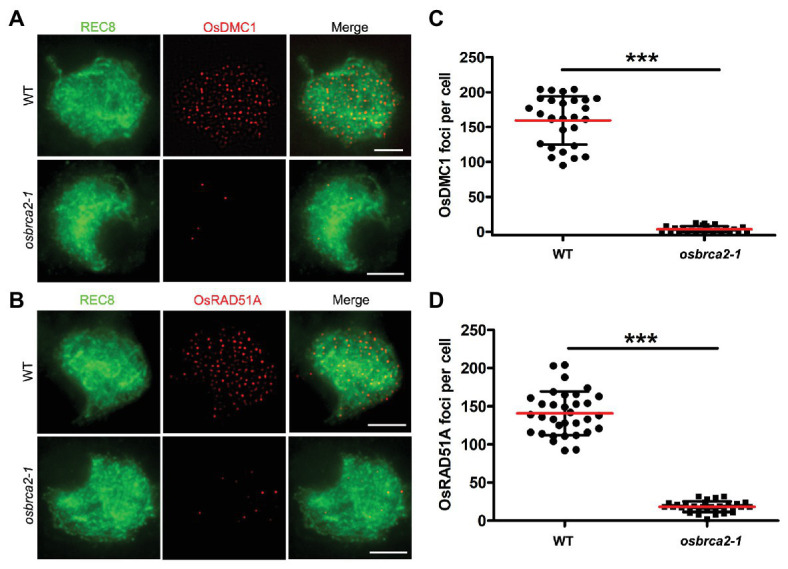
DMC1and RAD51A are not able to load on meiotic chromosomes in *osbrca2-1*. **(A)** Immunolocalization of DMC1 (red) and OsREC8 (green) in wild-type and *osbrca2-1* meiocytes at zygotene. **(B)** Immunolocalization of RAD51A (red) and OsREC8 (green) in wild-type and *osbrca2-1* meiocytes at zygotene. **(C)** Statistical analysis of the number of OsDMC1 foci per cell in wild type (*n* = 28) and *osbrca2-1* mutants (*n* = 26). **(D)** Statistical analysis of the number of OsRAD51A foci per cell in wild type (*n* = 33) and *osbrca2-1* mutants (*n* = 32). All values represent the mean ± SD, ^***^p < 0.0001, Student’s *t* tests. Bars = 2 μm.

During meiotic recombination, OsDMC1 and OsRAD51A are two key recombinases that play essential roles in crossover formation by promoting homology searches of the invading single strand and catalyzing strand exchange thereafter (Suwaki et al., 2011; Morozumi et al., 2013; Sansam and Pezza, 2015; Wang et al., 2016). In wild-type male meiocytes, OsDMC1 and OsRAD51A foci occurred at leptotene and peaked during zygotene (average 159.6, *n* = 28, and 140.8, *n* = 33, respectively). However, in *osbrca2-1* zygotene meiocytes, the number of DMC1 and OsRAD51A foci was substantially decreased (average 3.5, *n* = 26, ^***^p < 0.0001, Figures 7A,C; and 18.2, *n* = 32, ^***^p < 0.0001, Figures 7B,D), indicating that functional OsBRCA2 is indispensable for the localization of OsDMC1 and OsRAD51A onto chromosomes during meiosis. These results suggest that disruption of crossover formation in *osbrca2-1* is due to deficiency in OsDMC1 and OsRAD51A recruitment.

**Figure 8:**
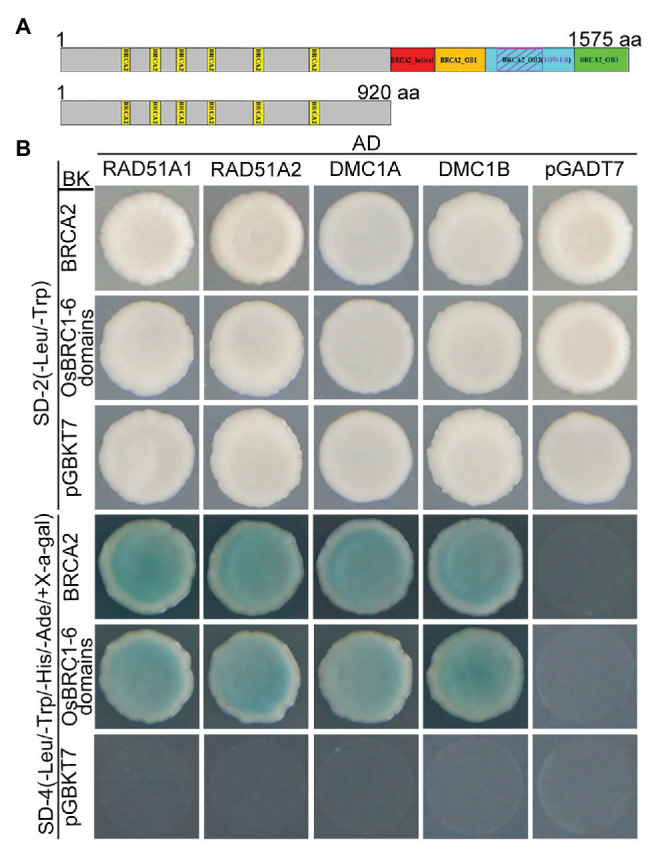
Interactions among OsBRCA2 and OsRAD51 paralogs. **(A)** A schematic diagram of OsBRCA2 and the truncation containing BRC1-6 motifs used in the following yeast two-hybrid assay is shown. **(B)** Yeast two-hybrid (Y2H) assay for interaction of OsBRCA2 with OsRAD51A1, OsRAD51A2, OsDMC1A, and OsDMC1B which verified on the SD-4 (-Leu/-Trp/-His/-Ade/+ X-α-gal) selection medium.

It has been previously reported that BRCA2 acts as a universal recombinase regulator and interacts with RAD51 and DMC1 homologs *via* BRC domains in many species (Fradet-Turcotte et al., 2016). To investigate whether OsBRCA2 also directly interacts with OsRAD51 or OsDMC1 in rice, yeast two-hybrid (Y2H) was performed. Rice contains two *RAD51* genes (*RAD51A1* and *RAD51A2*) as well as two *OsDMC1* genes (*OsDMC1A* and *OsDMC1B*). Y2H assays showed that both OsRAD51’s and OsDMC1’s interaction with the full length OsBRCA2 protein. Consistent with previous reports in other organisms, OsBRCA2 interacted with these proteins *via* its N-terminal OsBRC1-6 repeats (Figures 8A,B).

### The *osbrca2-1* Mutant Shows Hypersensitivity to Mitomycin C

Generally, mutants of genes involved in meiotic HR DSB repair show hypersensitivity to DNA mutagens, such as MMS, MMC, and UV irradiation (Waterworth et al., 2007; Chang et al., 2009; Kou et al., 2012; Zhang et al., 2015). Human pancreatic cancer cells expressing mutated BRCA2 lead to hypersensitivity from MMS treatment (Chen et al., 1998) and *hsbrca2* mutant embryos are also hypersensitive to γ-irradiation (Sharan et al., 1997). Similarly, *Arabidopsis brca2* double mutants are hypersensitive to the cross-linking agent MMC (Seeliger et al., 2012).

**Figure 9:**
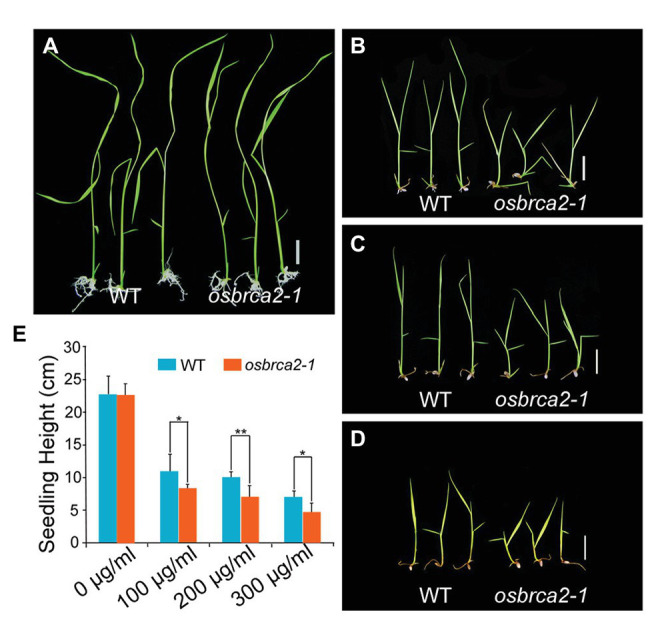
*Osbrca2-1* mutants are sensitive to mitomycin C (MMC). Phenotypes of wild-type and *osbrca2-1* seedlings growth in ½ MS medium at different MMC concentrations with 0 μg/ml **(A)**, 100 μg/ml **(B)**, 200 μg/ml **(C)**, and 300 μg/ml **(D)**. **(E)** Statistical analysis of the height of wild-type and *osbrca2-1* seedlings (*n* = 15, ^*^p < 0.05, ^**^p < 0.01, Student’s *t* tests). All values represent the mean ± SD. Bar = 2 cm.

*OsBRCA2* was broadly expressed not only in reproductive organs but also in vegetative organs, especially in leaves (Supplementary Figure S2). This suggests that although *OsBRCA2* is not required for normal vegetative growth, it may be involved in HR mediated DNA damage repair in somatic cells. To test whether *osbrca2-1* is sensitive to genotoxins like *hsbrca2* or *atbrca2*, we treated 5-day-old seedlings of the heterozygous *OsBRCA2-1*^+/−^ for 13 days with different concentrations of MMS or MMC. After treatment, the genotype of the seedlings was determined by PCR amplification and sequencing. There was no difference between *osbrca2-1* and wild-type seedlings when grown on 1/2 MS medium without genotoxins (*n* = 15, *p* = 0.84, Figures 9A,E). While grown on a medium containing 100–300 μg/ml MMC, *osbrca2-1* seedlings grew significantly slower than wild-type seedlings (*n* = 15, *p* < 0.05, Figures 9B,D,E; *p* < 0.01, Figures 9C,E) and after 20 days *osbrca2-1* mutants could not survive both low and high MMC concentrations. Moreover, about 30% (*n* = 15) *osbrca2-1*^+/−^ heterozygotes also died (Supplementary Figure S8). By contrast, the *osbrca2-1* plants showed an undistinguishable level of suppressed growth to that of the wild-type plants on culture medium supplemented with 50 μl/L and 100 μl/L MMS (*n* = 15, *p* > 0.05, Supplementary Figures S9A–C). In addition, both wild-type plants and *osbrca2-1* mutants could not survive after 150 μl/L MMS treatment (Supplementary Figure S9D). These results demonstrated that *osbrca2-1* plants were hypersensitive to the DNA damaging agent that causes interstrand cross-linking, indicating that *OsBRCA2* is essential for HR repair of DNA damage in somatic cells.

## Discussion

### OsBRCA2 Is Indispensable for Homologous Chromosome Pairing, Synapsis and DNA Repair in Rice Meiosis

In animals, BRCA2 plays essential roles in HR and HR-mediated DSB repair (O’Donovan and Livingston, 2010; Fradet-Turcotte et al., 2016). Compared with the extensive studies in animals, little is known about the function of BRCA2 in plants. Although orthologues of BRCA2 have been characterized in *Arabidopsis* (Siaud et al., 2004; Seeliger et al., 2012), it is yet unclear how widely these functions are conserved in other plants. In this study, our data reveals that mutations in rice *BRCA2* lead to severe meiotic defects and infertility, but have no obvious effects on normal vegetative growth, unless exposed to genotoxic agent MMC.

Homologous pairing, synapsis, and recombination are interdependent processes during meiosis in many organisms (Wang and Copenhaver, 2018). HR is essential for facilitating the formation of crossovers (COs) and accurate chromosome segregation. FISH analysis using 5*S* rDNA and OsCenH3 probes shows that homologous pairing was compromised in *osbrca2-1* (Figures 5C,D). Furthermore, the initiation of synapsis seemed normal but the synaptonemal complexes could not assemble successfully in the mutant, evidenced by the normal localization of PAIR3 while delayed depleting of PAIR2 and the failure of ZEP1 assembly (Figures 6A,B) at pachytene. At diakinesis, univalents instead of bivalents were observed in *osbrca2*, causing random segregation of homologous chromosomes at anaphase I. These results suggest that OsBRCA2 acts at an early stage of HR by mediating DSB dependent chromosome contacts, which is critical to establish physical linkage and ensure proper separation of homologous chromosomes.

The meiotic defects in *osbrca2* are consistent with those observed in mammals and *Arabidopsis* for *brca2* mutants, but in contrast to that of *Caenorhabditis elegans*, in which no homolog alignment or SC assembly aberrance is detected (Martin et al., 2005). The *atbrca2a/b* double mutant is able to set a few seeds when pollinated with wild type pollen grains (Seeliger et al., 2012), indicating that deletion of both *AtBRCA2A/B* does not lead to the complete loss of female gametophyte function. However, the male mice hypomorphic *brca2* mutant is completely sterile, but in females some oocytes undergo meiosis and fertilization that finally develop into embryos (Sharan et al., 2004). By contrast, *osbrca2-1* plants could not set seeds either used as maternal or paternal recipients. These observations suggest a functional divergence for the necessity of BRCA2 in different species and a different requirement of BRCA2 in male and female meiocyte development.

### OsBRCA2 Is Essential for DNA Repair in Both Meiotic and Somatic Cells

Homologous recombination is one of the most effective mechanisms for DSB repair, which guarantees accurate repair of DSBs within homologous sequences (Cloud et al., 2012). BRCA2 plays a central role in HR-mediated DSB repair in animals and *Arabidopsis* (Gudmundsdottir and Ashworth, 2004; Thorslund and West, 2007). The normal localization of γH2AX, COM1, RPA1c and RPA2c in male meiocytes of *osbrca2-1* suggests that *OsBRCA2* is dispensable for DSB production and its end processing (Supplementary Figure S6). However, chromosome entanglements and fragments are observed at diakinesis and later stages (Figure 4). Furthermore, these abnormalities are completely eliminated by the mutation in *OsSDS*, a key factor required for meiotic DSB generation in rice (Supplementary Figure S5), indicating that *OsBRCA2* plays a conserved role in programmed meiotic DSB repair.

BRCA2 is not only essential for meiotic DSB repair but also important for DSB repair in mitotic cell. Animals carrying a complete loss-of-function allele of *BRCA2* are embryo lethal, suggesting cell cycle arrest and cell death triggered by the accumulating DNA damages and increased genomic instability. Hypomorphic *brca2* mutants also showed hypersensitivity to DNA-damage agents, such as MMS, MMC, UV irradiation, which is a common feature of mutants defective in HR repair (Chen et al., 1998, 1999; Yuan et al., 1999; Yu et al., 2000; Masson et al., 2001; Wiese et al., 2002; Ohashi et al., 2005; Meyer et al., 2017). In *Arabidopsis*, both *Atbrca2a* and *Atbrca2b* single mutants and the double mutant are viable and appear healthy under normal growth conditions, while being hypersensitive to MMC (Wang et al., 2010b; Seeliger et al., 2012). Our study shows that *osbrca2-1* seedlings are also hypersensitive to MMC (Figure 9), which introduces DNA interstrand cross-linking (Lehoczky et al., 2007). Interestingly, the heterozygotes of *osbrca2-1* seedlings also exhibit higher ratio of death after treatment by low or high concentrations of MMC for 20 days (Supplementary Figure S7), indicating that adequate expression of *BRCA2* is essential for DNA repair in rice somatic cells.

MMS causes damage by methylating DNA on the N7-deoxyguanine and N3-deoxyadenine (Vázquez et al., 2008). It has been shown that a truncated BRCA2 strain in mouse exhibits a striking increased sensitivity to MMS treatment. However, *osbrca2-1* and wild-type seedlings displayed a similar sensitivity to MMS at a series of concentrations (Supplementary Figure S8). Different phenotypic outcomes of *brca2* mutants to MMS in rice and mouse are probably due to DNA pathway choices. Another possibility is that there might be tissue or developmental stage specific sensitivity to genotoxic agents. For example, mouse embryo fibroblasts bearing the *Brca2^Tr/Tr^* mutation do not exhibit sensitivity to MMC, while *Brca2^Tr/Tr^* lymphoid cells are highly sensitive to MMC (Patel et al., 1998). These results confirmed the conserved function of BRCA2 in repairing DNA damage among different species.

### The Role of *OsBRCA2* in HR Mediated DNA Repair

In humans, BRCA2 interacts with RAD51 *via* BRC repeats and the TR2 domain located on the C-terminus. The BRC repeats of HsBRCA2 can also directly interact with DMC1 to stimulate single-strand invasion between homologous chromosomes. Besides, the PhePP domain of HsBRCA2 is specially bound by DMC1, but not for RAD51. (Davies and Pellegrini, 2007; Thorslund et al., 2007; Fradet-Turcotte et al., 2016; Martinez et al., 2016). The distinct interacting motifs on BRCA2 with RAD51 and DMC1 confer the universal functions of BRCA2 in regulating actions of two recombinases in both germinal and somatic cells. In *Arabidopsis*, there are two isoforms of BRCA2. Both AtBRCA2 can interact with AtRAD51 and AtDMC1 *in vitro* (Siaud et al., 2004; Dray et al., 2006) and are required for their recruitment to the chromosomes in meiocytes (Seeliger et al., 2012).

Our results show that both the full length and the truncated OsBRCA2 containing the six BRC repeats are capable of interacting with the two isoforms of OsRAD51A and OsDMC1 (Figure 8), suggesting a conserved function of the BRC repeats in OsBRCA2. Our results further reveal that both OsRAD51A and OsDMC1 foci are dramatically reduced in *osbrca2-1* compared with wild type (Figure 7), confirming that the correct localization of OsRAD51As and OsDMC1 onto meiotic chromosomes also depends on a functional BRCA2 in rice. In contrast to *Arabidopsis*, OsDMC1 is not required for homologous chromosome pairing in rice (Wang et al., 2016). Previous reports have demonstrated the important role of RAD51 in homology searching and pairing in many organisms (Bishop, 1994; Pawlowski et al., 2003; Li et al., 2004). Thus, the abnormality in homologous pairing may mainly be due to the failure in recruiting OsRAD51A’s, although this is yet to be investigated in rice. Taken together, our study provides evidence for the conserved function of rice BRCA2 as a central regulator of RAD51 and DMC1 recruitment, to facilitate single-strand invasion during HR and HR-mediated DSB repair.

## Data Availability Statement

The original contributions presented in the study are included in the article/Supplementary Material, further inquiries can be directed to the corresponding author.

## Author Contributions

WL conceived the project. RF, CW, HS, and JZ performed experiments. RF wrote the article. WL and JH supervised and complemented the writing. All authors contributed to the article and approved the submitted version.

### Conflict of Interest

The authors declare that the research was conducted in the absence of any commercial or financial relationships that could be construed as a potential conflict of interest.
