# Supplementary material for: Rice OsBRCA2 Is Required for DNA Double-Strand Break Repair in Meiotic Cells
Source: Front Plant Sci. 2020 Nov 16;11:600820. doi: 10.3389/fpls.2020.600820 (PMC7701097; doi:10.3389/fpls.2020.600820)
Supplement: Supplementary file 1 [file Table_1.docx]

**Supplemental Table 1. Primers Used in This Study**

| **Primer name** | **Sequence （5'-->3'）** | **Objective** |
| --- | --- | --- |
| SHY101-2-2F | CCAAACAGAGTCGAGTATGCCACG | Fine mapping |
| SHY101-2-2R | CGGACGATCAAAGTTGAGCACAGA | Fine mapping |
| SHY102-3-1F | AACACCATCCAGAAAAAGAAAACG | Fine mapping |
| SHY102-3-1R | TCTCTCACCTCCCTGACACTAAACT | Fine mapping |
| OsBRCA2-CRISPR-1F | TGGCGAGCAAGGGCTCAATTCAGA | *osbrca2-2* CRISPR |
| OsBRCA2-CRISPR-1R | AAACTCTGAATTGAGCCCTTGCTC | *osbrca2-2* CRISPR |
| OsBRCA2-CRISPR-2F | TGGCGGGGTTAGGGAGGCCTGTTG | *osbrca2-3* CRISPR |
| OsBRCA2-CRISPR-2R | AAACCAACAGGCCTCCCTAACCCC | *osbrca2-3* CRISPR |
| OsBRCA2-CRISPR-3F | TGGCGCTTCACTATGTGGGTGGGC | *osbrca2-4* CRISPR |
| OsBRCA2-CRISPR-3R | AAACGCCCACCCACATAGTGAAGC | *osbrca2-4* CRISPR |
| OsBRCA2 ide-F | TTTGGAGGGACAAACAAATC | Identification for *osbrca2-1* |
| OsBRCA2 ide-R | TGGGAGGAAAGTTCATTACA | Identification for *osbrca2-1* |
| OsBRCA2-CRISPR ide-1F | TGTATGTATTGCAGGGCAAC | Identification for *osbrca2-2* |
| OsBRCA2-CRISPR ide-1R | GGCCTAACCAATCCAGTATG | Identification for *osbrca2-2* |
| OsBRCA2-CRISPR ide-2F | GCCTGACCAATCTCTGATTT | Identification for *osbrca2-3* |
| OsBRCA2-CRISPR ide-2R | ACCATGTACCTTGCATTGAA | Identification for *osbrca2-3* |
| OsBRCA2-CRISPR ide-3F | AGACGACGACAACAGTGAGA | Identification for *osbrca2-4* |
| OsBRCA2-CRISPR ide-3R | CGATAGGTGCCATTTATGTG | Identification for *osbrca2-4* |
| OsBRCA2-Ab-F | ATGGCTGATATCGGATCCGAACCATTGTTCCAAACTGGTTCAG | Antibody of OsBRCA2 |
| OsBRCA2-Ab-R | GTGGTGGTGGTGGTGGTGCTCTTAGGCACAGCCATCATCTGAGTGAG | Antibody of OsBRCA2 |
| OsRAD51-Ab-F | ATGGCTGATATCGGATCCGAAATGTCGACGTCGGCGG | Antibody of OsRAD51 |
| OsRAD51-Ab-R | GTGGTGGTGGTGGTGGTGCTCTTATTGATGATCAGTATTATAAGCTCTG | Antibody of OsRAD51 |
| OsBRCA2 qRT-1F | GATGATAACGATGAAGGGGC | qRT-PCR Analysis |
| OsBRCA2 qRT-1R | CCAGATTTTGTGGCGGAGTG | qRT-PCR Analysis |
| Actin-F | ACCCAAGAATGCTAAGCCAAGAG | qRT-PCR Analysis |
| Actin-R | ACTTTGTCCACGCTAATGAAGAAAC | qRT-PCR Analysis |
| OsBRCA2-BK-1F | CTGTATCGCCGGCCAATCCGGCCATGCAGAGGAGGTGGCAGGTGTGG | Y2H |
| OsBRCA2-BK-1R | GCCGCTGCAGGGCCTCTAGGATCCATAAGTACTTGGTTTTCA | Y2H |
| OsBRCA2-BK-2F | CCAAGTACTTATGGATCC ACATCAGCATTTTGCATTTTC | Y2H |
| OsBRCA2-BK-2R | GTTATGCGGCCGCTGCAGTCAGCCACCACTGTCACCAACTAT | Y2H |
| OsBRC1-6 domains-BK-F | AGGAGGACCTGCATATGCAGAGGAGGTGGCAGGTGT | Y2H |
| OsBRC1-6 domains-BK-R | TGCAGGTCGACGGATCCAGGATAGCATGAAGATAGCT | Y2H |
| OsDMC1A-AD-F | AGGAGGACCTGCATATGGCGCCGTCCAAGCAGTACAG | Y2H |
| OsDMC1A-AD-R | TGCAGGTCGACGGATCCTCAGTCTTTCGCATCCATTAT | Y2H |
| OsDMC1B-AD-F | AGGAGGACCTGCATATGGCGCCGTCCAAGCAGTACGA | Y2H |
| OsDMC1B-AD-R | TGCAGGTCGACGGATCCTCAGTCTTTCGCATCCATTAT | Y2H |
| OsRAD51A1-AD-F | CAGATTACGCTCATATGTCGACGTCGGCGGCGGCGTCG | Y2H |
| OsRAD51A1-AD-R | CGAGCTCGATGGATCCTCAATCCTTGACATCTGCAAC | Y2H |
| OsRAD51A2-AD-F | CAGATTACGCTCATATGTCGTCGTCGGGTGCGGCTGCG | Y2H |
| OsRAD51A2-AD-R | CGAGCTCGATGGATCCTCAGTCCTTAACATCTGTGAC | Y2H |
