# Supplementary material for: Rice OsBRCA2 Is Required for DNA Double-Strand Break Repair in Meiotic Cells
Source: Front Plant Sci. 2020 Nov 16;11:600820. doi: 10.3389/fpls.2020.600820 (PMC7701097; doi:10.3389/fpls.2020.600820)
Supplement: Supplementary file 2 [file Data_Sheet_1.pdf]

Supplementary Images

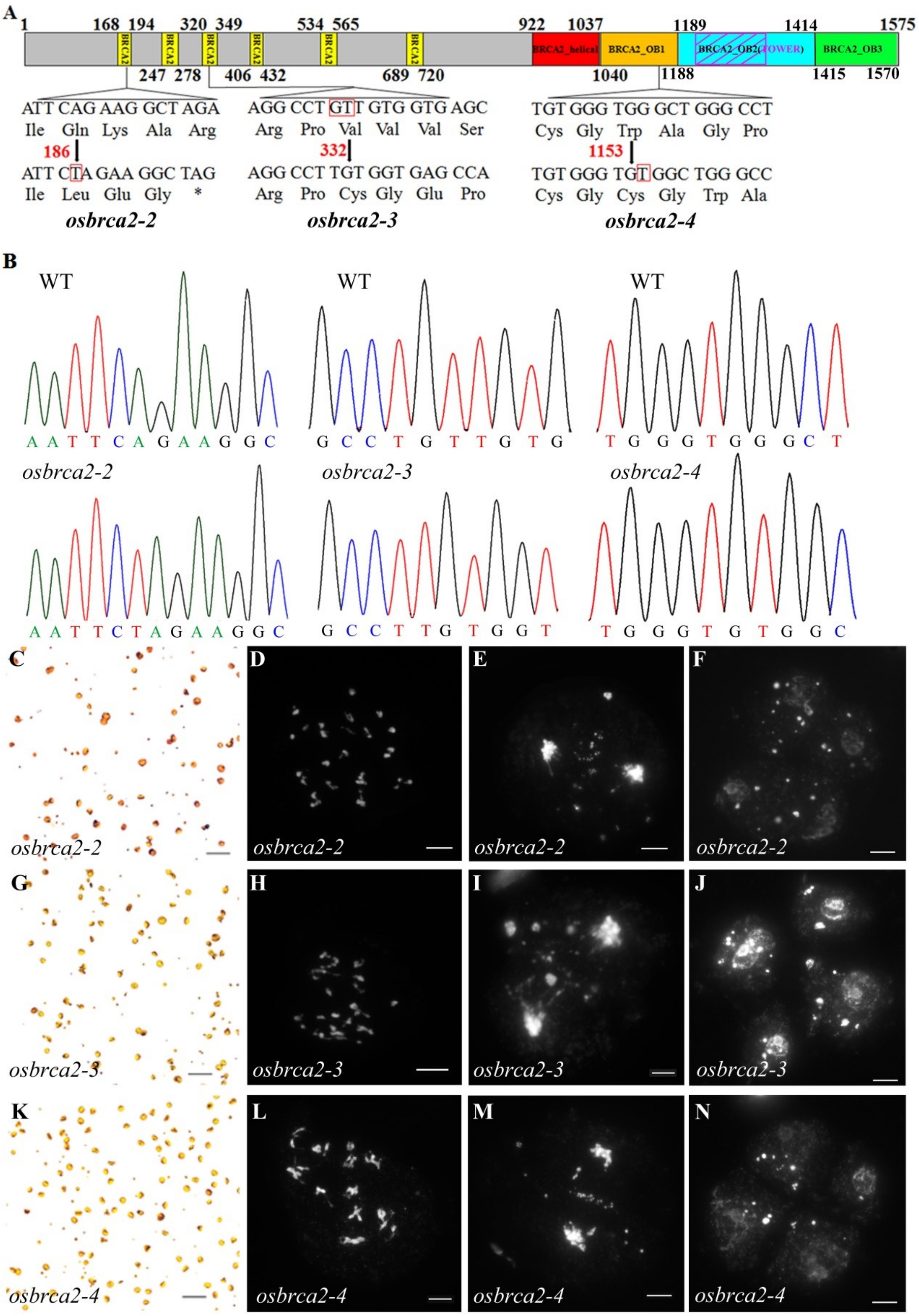

Supplemental Figure S1. Mutations and phenotypes of *OsBRCA2* CRISPR lines.

(A) A schematic diagram of the OsBRCA2 protein. Arrows indicated the mutation sites. *Osbrca2-2* and *osbrca2-4* had a T insertion, which caused a frame shift from 186 aa and 1153 aa respectively and premature translation termination. *Osbrca2-3* had two base pairs (GT) deletion,

and caused a frame shift from 332 aa and premature translation termination.

(B) Sequencing results of the *OsBRCA2* gene in wild-type and *OsBRCA2* CRISPR lines.

(C, G, K) I_2-KI staining of the pollen grains from the anther of *osbrca2-2 osbrca2-3* and *osbrca2-4*. **(D - F)**, **(H - J)**, and **(L - N)** *osbrca2-2 osbrca2-3* and *osbrca2-4* PMCs at the diakinesis, anaphase I and tetrad stage respectively. 100 μm in **(C**, **G, K)**, 5μm in **(D - F)**, **(H - J)**, and **(L - N)**.

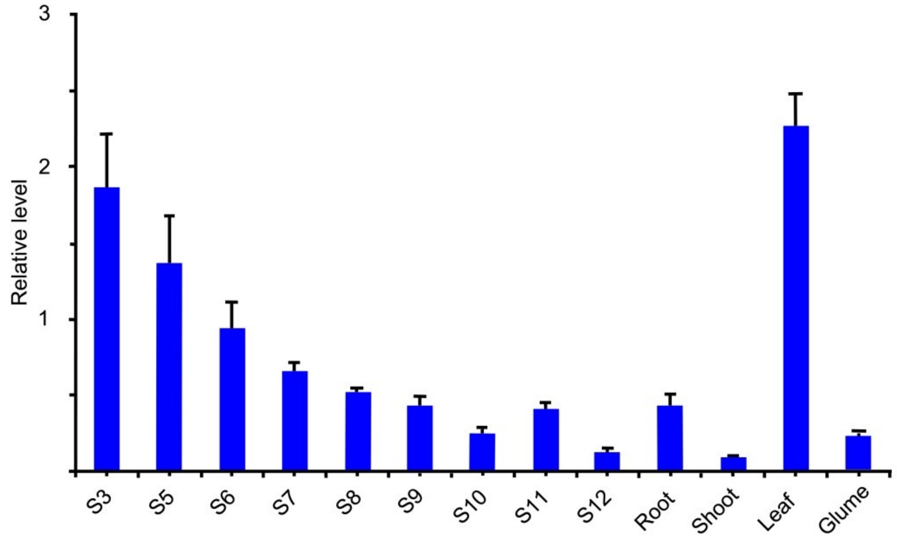

Supplemental Figure S2. Spatiotemporal analysis of *OsBRCA2* transcription by qRT–PCR. *OsBRCA2* expression pattern in the wild-type plant at different development stages (S3 to S12) and tissues (anther, root, shoot, leaf, and glume). All reactions were performed in three independent biological replicates with three technical repeats each for statistical analysis. The gene expression was calculated by the $2^{-\Delta\Delta Ct}$ method.

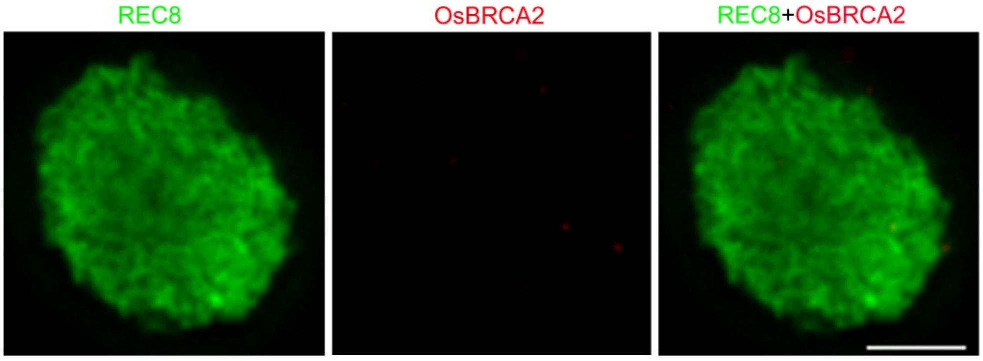

Supplemental Figure S3. Dual immunolocalization assay of OsBRCA2 (red) and REC8 (green) at zygotene in *osbrca2-1* male meiocytes. Bar = 5 μm.

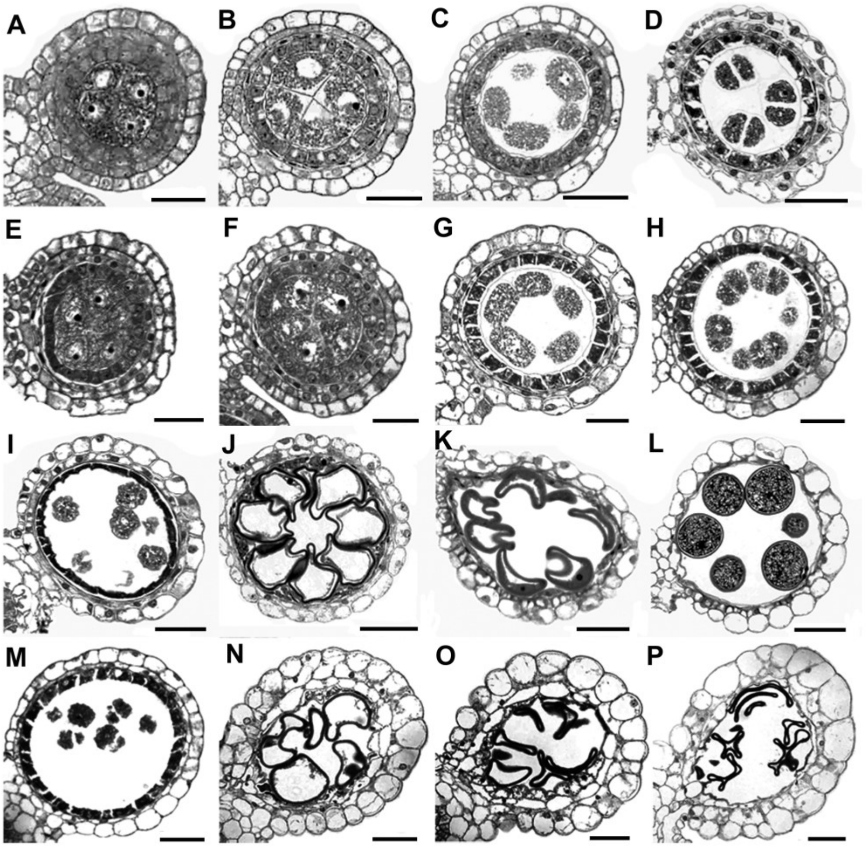

Supplemental Figure S4. Transverse section observation of the anther development in wild-type and *osbrca2-1*.

Anther locules from the wild-type **(A - D, I - L)** and *osbrca2-1* **(E - H, M - P)**.

(A, E) Stage 6 (the pre-meiosis stage).

(B, F) Stage 7 (the MMC stage).

(C, G) Stage 8a (the dyad stage).

(D, H) Stage 8b (the tetrad stage).

(I, M) Stage 9 (the young microspore stage).

(J, N) Stage 10 (the vacuolated pollen stage).

(K, O) Stage 11 (2-uncleate stage).

(L, P) Stage 12 (the mature pollen stage). Bars =20 μm.

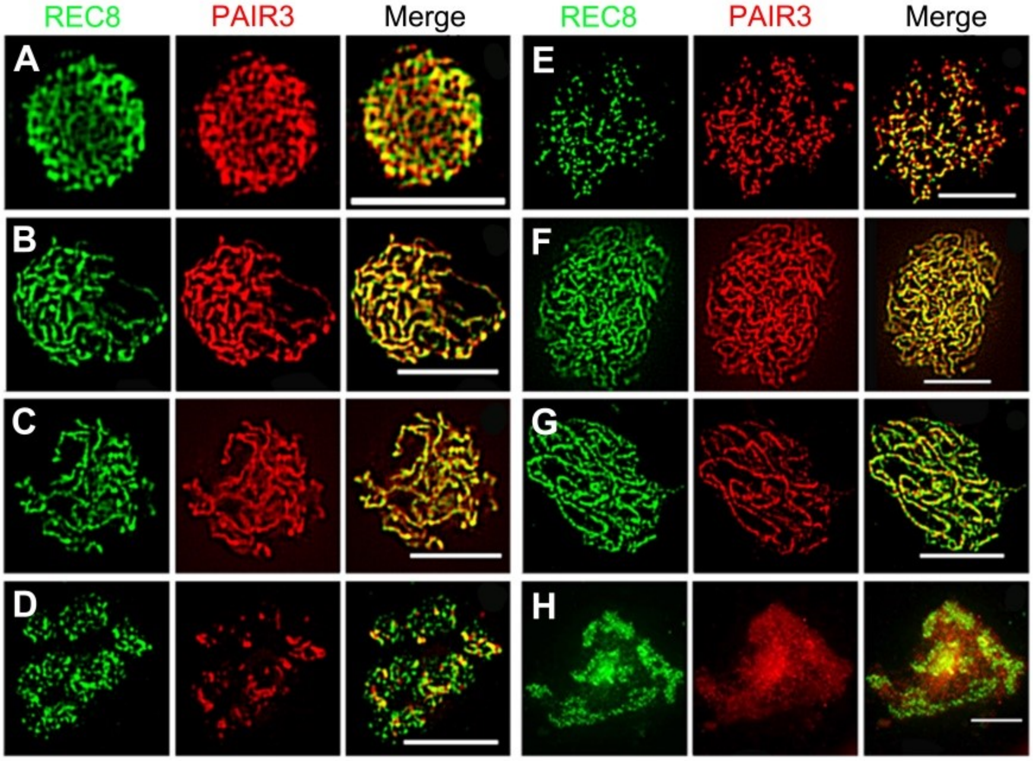

Supplemental Figure S5. Dual immunolocalization of REC8 (green) and PAIR3 (red) in wild-type (A - D) and *osbrca2-1* (E - H).

(A, E) Zygotene. **(B, F)** Early pachytene. **(C, G)** Pachytene. **(D, H)** Diakinesis. Bars=5 µm.

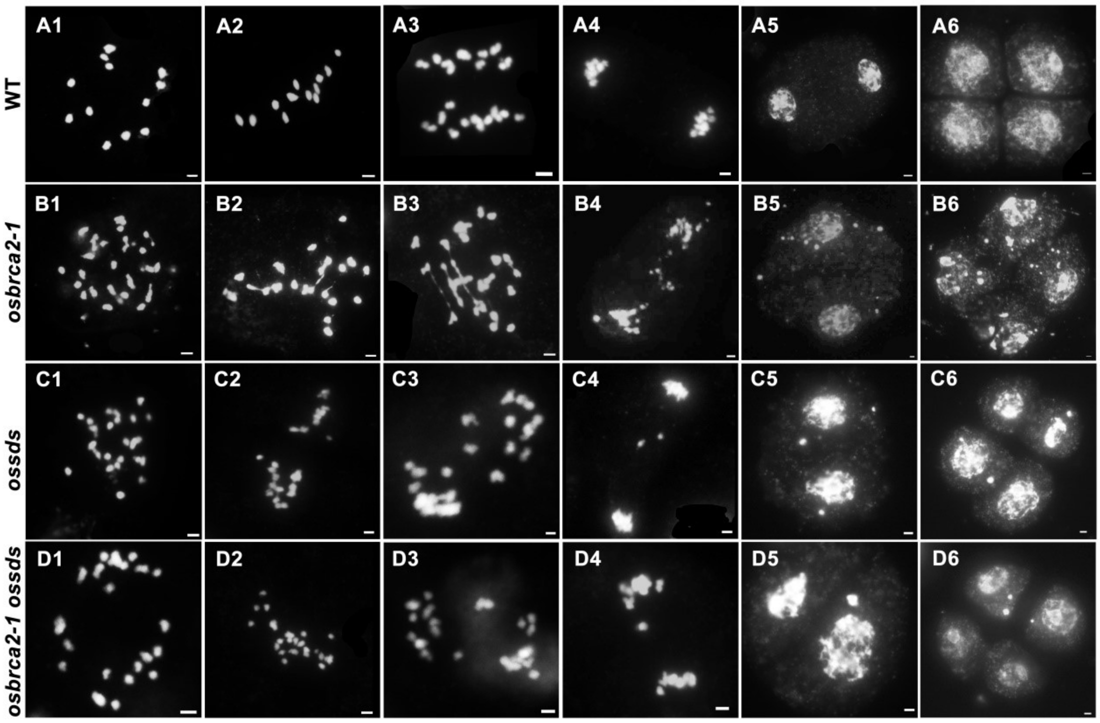

Supplemental Figure S6. Meiotic chromosome behaviors of male meiocytes among the wild type, *osbrca2-1*, *ossds*, and *osbrca2-1 ossds* double mutant.

(A1 - D1) Diakinesis. (A2 - D2) Metaphase I. (A3 - D3) Anaphase I. (A4 - D4) Telophase I. (A5 - D5) Dyad. (A6 - D6) Tetrad. Bars = 2 μm.

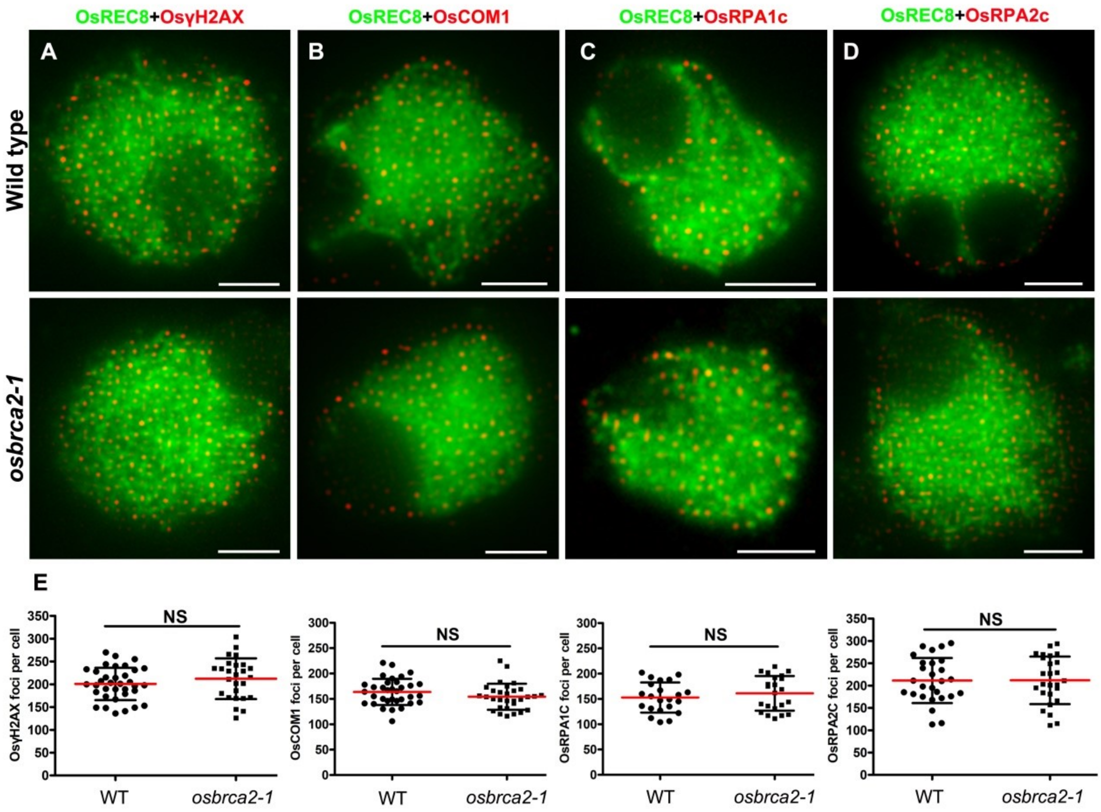

Supplemental Figure S7. OsBRCA2 is dispensable for DSB production and its end processing.

Dual Immunolocalization of OsREC8 (green) and OsγH2AX **(A)**, OsCOM1 **(B)**, OsRPA1c **(C)**, and OsRPA2c **(D,** red**)** respectively in wild type and *osbrca2-1* zygotene meiocytes. **(E)** Statistical analysis of the number of OsγH2AX, OsCOM1, OsRPA1c, and OsRPA2c foci per cell between wild type (n = 35, 34, 22, 27, respectively) and *osbrca2-1* mutants (n = 26, 30, 22, 26, respectively) revealed no significant differences (NS, P >0.05, Student's t tests). The values represent the average foci number of OsγH2AX, OsCOM1, OsRPA1c, and OsRPA2c in wild type and *osbrca2-1* zygotene and "n" represents the cell number counted. All values represent the mean ± SD. Bars = 2 μm.

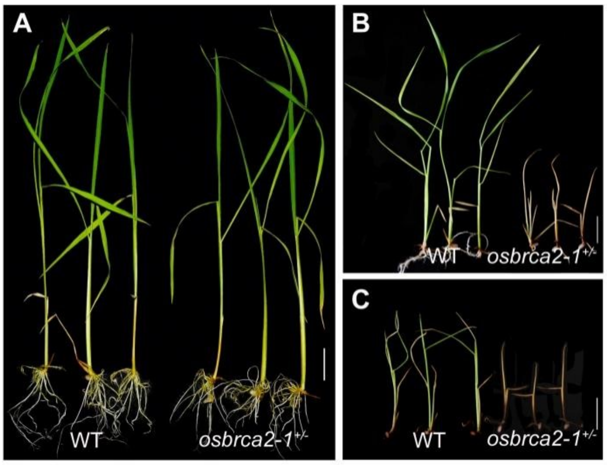

Supplemental Figure S8. The *osbrca2-1*$^{+/–}$ heterozygotes died after 20-day MMC treatment. Performance of wild-type and *osbrca2-1*$^{+/–}$ heterozygotes growth in ½MS medium at different MMC concentrations with 0 µg/ml **(A)**, 100 µg/ml **(B)**, and 300 µg/ml **(C)** after growing for 20 days. About 30% (n=15) *osbrca2-1*$^{+/–}$ heterozygotes died both low and high MMC concentrations medium. Bars=2 cm.

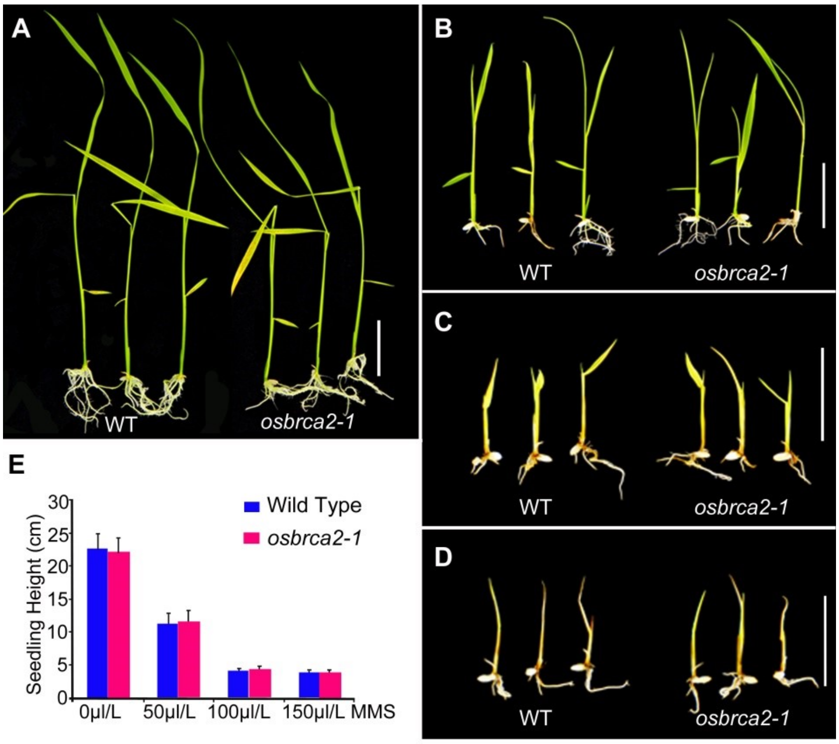

Supplemental Figure S9. The *osbrca2-1* seedlings are not hypersensitive to MMS.

(A), **(B)**, **(C)** and **(D)** Phenotypes of wild-type and *osbrca2-1* seedlings grown on one-half MS (Murashige and Skoog) supplemented with different concentrations of methyl methanesulfonate (MMS) from 0 μl/L to 150 μl/L. **(E)** Statistical analysis of the height of wild-type (WT) and *osbrca2-1* seedlings revealed no significant differences (n=15, P>0.05, Student's t tests). All values represent the mean ± SD. Bars=4 cm.
